# Gut Microbial Community and Host Thermoregulation in Small Mammals

**DOI:** 10.3389/fphys.2022.888324

**Published:** 2022-04-11

**Authors:** Xue-Ying Zhang, De-Hua Wang

**Affiliations:** ^1^ State Key Laboratory of Integrated Management of Pest Insects and Rodents, Institute of Zoology, Chinese Academy of Sciences, Beijing, China; ^2^ School of Life Sciences, Shandong University, Qingdao, China; ^3^ CAS Center for Excellence in Biotic Interactions, University of Chinese Academy of Sciences, Beijing, China

**Keywords:** gut microbiota, norepinephrine (NE), small mammals, thermoregulation, thyroid hormones

## Abstract

The endotherms, particularly the small mammals living in the polar region and temperate zone, are faced with extreme challenges for maintaining stable core body temperatures in harsh cold winter. The non-hibernating small mammals increase metabolic rate including obligatory thermogenesis (basal/resting metabolic rate, BMR/RMR) and regulatory thermogenesis (mainly nonshivering thermogenesis, NST, in brown adipose tissue and skeletal muscle) to maintain thermal homeostasis in cold conditions. A substantial amount of evidence indicates that the symbiotic gut microbiota are sensitive to air temperature, and play an important function in cold-induced thermoregulation, via bacterial metabolites and byproducts such as short-chain fatty acids and secondary bile acids. Cold signal is sensed by specific thermosensitive transient receptor potential channels (thermo-TRPs), and then norepinephrine (NE) is released from sympathetic nervous system (SNS) and thyroid hormones also increase to induce NST. Meanwhile, these neurotransmitters and hormones can regulate the diversity and compositions of the gut microbiota. Therefore, cold-induced NST is controlled by both Thermo-TRPs—SNS—gut microbiota axis and thyroid—gut microbiota axis. Besides physiological thermoregulation, small mammals also rely on behavioral regulation, such as huddling and coprophagy, to maintain energy and thermal homeostasis, and the gut microbial community is involved in these processes. The present review summarized the recent progress in the gut microbiota and host physiological and behavioral thermoregulation in small mammals for better understanding the evolution and adaption of holobionts (host and symbiotic microorganism). The coevolution of host-microorganism symbionts promotes individual survival, population maintenance, and species coexistence in the ecosystems with complicated, variable environments.

## Introduction

The endotherms regulate thermogenesis and/or heat dissipation to maintain relatively high, stable core body temperatures (*T*_b_, around 37°C in many species) in the fluctuating environments. Small mammals living in the polar region and temperate zone, due to with high ratios of body surface area to volume, are faced with extreme challenges in harsh winter with the characteristics of low temperatures and food shortage. During evolution, these mammals have developed multiple strategies, by adjusting morphological, physiological, and behavioral phenotypes, to adapt to variable environments, especially fluctuations of air temperatures (*T*_a_). A crucial physiological strategy for small mammals to cope with *T*_a_ fluctuations is changing metabolic rate for survival during seasonal acclimatization.

Some mammalian species are able to decrease metabolic rate and *T*_b_, and enter torpor (such as *Phodopus sungorus* and *P. rovorovskii*) or hibernation (such as *Spermophilus dauricus*) to save energy and survive in winter (McNab, 2002; Chi et al., 2016; Ren et al., 2022). However, the non-hibernating small mammals (e.g., *Lasiopodomys brandtii* and *Meriones unguiculatus*) must increase metabolic rate including obligatory thermogenesis (basal/resting metabolic rate, BMR/RMR, with a difference of fasting for the former and no fasting for the latter before measurement) in the liver and other metabolic organs, and regulatory thermogenesis (mainly nonshivering thermogenesis, NST) in brown adipose tissue (BAT) and skeletal muscle to maintain thermal homeostasis in winter or during cold acclimation (Cannon and Nedergaard, 2004; Zhang and Wang, 2006; Nowack et al., 2017; Bal et al., 2018). Both the sympathetic nervous system (SNS) and thyroid hormones are required for regulating thermogenesis and *T*_b_ (Cannon and Nedergaard, 2004; Mullur et al., 2014). These non-hibernating mammals need consume more food to compensate for the high energy expenditure due to cold-induced thermogenesis (Zhang and Wang, 2006). In fact, food resource is not available all the time and is even of severe shortage in wild winter. Therefore, these small mammals have also evolved behavioral strategies, for example huddling and coprophagy, to decrease energy expenditure and increase energy harvest.

In addition to the physiological and behavioral strategies of the rodents themselves, a myriad of evidence indicates that the complex communities of symbiotic microorganisms residing in the gastrointestinal tract of animals can largely affect host phenotypes and fitness, such as energy metabolism, thermogenesis, immune function, endocrine activity and neuronal functions (Fetissov, 2017; Rastelli et al., 2019). This microbial community is specific to each host individual in spite of the existence of multiple bacterial taxa shared by the majority of hosts. The gut microbiota can degrade dietary fiber (nondigestible carbohydrates) into short-chain fatty acids (SCFAs, mainly acetate, propionate, and butyrate) and vitamins, and also contribute to metabolizing primary bile acids into secondary bile acids (SBAs) by enhancing deconjugation, dehydrogenation, and dehydroxylation via bacterial enzymes (Ridlon et al., 2006; Rastelli et al., 2019). It is verified that both SCFAs and SBAs are involved in stimulating gut hormone secretion and regulating appetite and thermogenesis. These metabolites and byproducts of microbiota can also directly interact with the enteric nervous system (ENS) and provide a link between the gut microbiota and host physiology and behavior (Worthmann et al., 2017). The experiments of gut microbiota transplant revealed that alteration in the gut microbiota induced variations in host phenotypes, further suggesting that the specific gut microbes are of importance for host fitness (Gould et al., 2018). Moreover, microbial communities codiversified with their host species, implying coevolution between the gut microbiota and their hosts (Ley et al., 2008; Groussin et al., 2017). Therefore, the evolution theory of host-microorganism symbioses assumes that microbial colonization in the mammalian intestinal tracts should be an evolution-driven process benefiting fitness of both sides. In this review, we summarize recent evidence about the physiological and behavioral strategies for maintaining *T*_b_ in response to changing *T*_a_ and the functions of the gut microbiota in host thermoregulation in small mammals for better understanding the evolution and adaption of holobionts.

### Environmental Cues and Nonshivering Thermogenesis

Environmental cues play crucial roles in affecting animal’s physiological and behavioral plasticity. Among these cues, *T*_a_, due to its seasonal, daily and sometimes irregular fluctuations, is remarkable in affecting thermogenesis, energy balance and survival in small mammals living in the arctic and temperate regions. Small mammals change metabolic rate in response to *T*_a_ fluctuations. The metabolic rate, measured in the resting, awake, and postabsorptive state and named as the BMR, is kept at a constant, minimal level within a specific temperature range, which is defined as the thermoneutral zone (TNZ) (Gordon, 2012). The idea of a thermoneutral “zone” has been questioned and a thermoneutral point (TNP) was proposed, blow which metabolic rates increase and above which *T*_b_ increases based on studies in mice (Škop et al., 2020). In wide rodents such as Mongolian gerbils, there is a temperature range within which both RMR and *T*_b_ were kept relatively stable (Pan et al., 2014; Zhang et al., 2016; Ding et al., 2018). Energy expenditure (oxygen consumption) increases, due to cold-induced thermogenesis at *T*_a_ below the TNZ, and due to a Q10 effect (Mikane et al., 1999) or cooling mechanisms related with water balance and osmotic regulation at *T*_a_ above the TNZ (Kasza et al., 2019). Since thermal constraints across geographic gradients, each mammal species has formed species-specific TNZ, which may reflect the differences in the thermal biology among species (Bozinovic et al., 2014).

In eutherian mammals, BAT, has evolved as a specialized thermogenic organ and confers mammals an evolutionary consequence to survive the cold stress (Oelkrug et al., 2015). BAT depots are predominantly located in the intrascapular, dorso-cervical, subscapular and axillary regions, and sporadically in the pericardial and perirenal regions in rodents (Smith and Roberts, 1964; Symonds et al., 2015). The adipocytes in BAT contain dense mitochondria for high oxidative capacity and energy production, and dense vascularization helpful to transport nutrient and heat, as well as multilocular fat droplets to be mobilized into free fatty acids as fuel rapidly (Cannon and Nedergaard, 2004). Cold-induced BAT thermogenesis is mediated through stimulation of β3-adrenergic receptors (β3-AR)-dependent protein kinase A (PKA) signaling and activation of uncoupling protein 1 (UCP1). UCP1 is almost exclusively located in the inner mitochondrial membrane of brown adipocytes, and plays the function of uncoupling the proton motive force from ATP production and dissipating the electrochemical energy as heat (Cannon and Nedergaard, 2004). It was observed that BAT mass, mitochondrial protein content, cytochrome c oxidase (COX) activity and UCP1 expression increased, during cold exposure or cold acclimation in non-hibernating small mammals, such as in Brandt’s voles (*Lasiopodomys brandtii*), Mongolian gerbils root voles (*Microtus oeconomus*) and plateau pikas (*Ochotona curzoniae*) (Wang et al., 2006; Zhang and Wang, 2006; Li and Wang, 2007). Cold-induced increases in sympathetic activation and UCP1 expression (browning) in white adipose tissue (WAT) can also help animals defense cold and maintain *T*_b_ (Bai et al., 2015; Shin et al., 2017). Our previous data confirmed that cold-induced phenotypic variations were initiated by increased release of norepinephrine (NE) and activation of cAMP-PKA-pCREB signaling pathway in BAT (Bo et al., 2019). Cold-induced lipolysis (catabolism of triglycerides) and glucose uptake via glucose transporter (GLUT4) in adipocytes provides the availability of free fatty acids and glucose as the sources of fuel for BAT thermogenesis (Townsend and Tseng, 2014; Garretson et al., 2016). Therefore, BAT plays its roles in regulating thermogenesis, and fatty acid and glucose metabolism.

**FIGURE 1:**
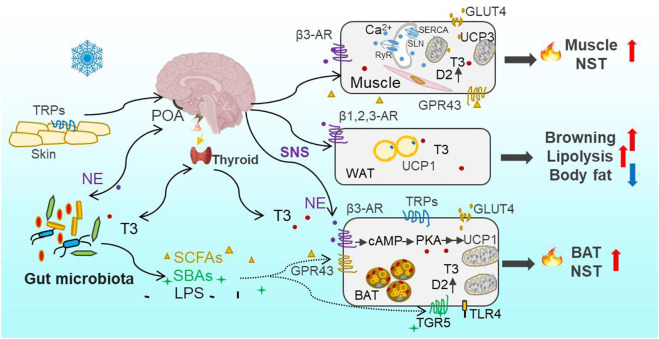
Overview of cold-induced thermogenesis through Thermo-TRPs—SNS—gut microbiota—thermogenesis axis and thyroid—gut microbiota—thermogenesis axis. Cold is sensed by the thermosensitive transient receptor potential channels (Thermo-TRPs) on the skin and in the brain and other organs. Cold signal is transmitted through the sensory afferent nerve to the preoptic area (POA) in the hypothalamus and other thermoeffector efferent circuits, and leads to the activation of the sympathetic nervous system (SNS) to release norepinephrine (NE) and stimulate mitochondrial uncoupling protein 1 (UCP1) through β3 adrenergic receptors (*β*3-AR) and activation of PKA signaling pathway in brown adipose tissue (BAT). In parallel with the direct effect of NE on thermogenesis, the gut microbiota community is reconstructed through sensing cold signal or neuroactive signal of NE and, consequently, leads to increases in bacterial metabolites (short-chain fatty acids, SCFAs) and byproducts (secondary bile acids, SBAs). Both SCFAs and SBAs, via acting on their respective G protein-coupled receptors GPR43 and TGR5, can stimulate BAT nonshivering thermogenesis (NST). Moreover, the circulating bioactive tri-iodothyronine (T3) level increases after the brain receives cold signal, and promotes obligatory (basal metabolic rate, BMR) and regulatory thermogenesis. T3-induced thermogenesis is also mediated by bacterial metabolites (SCFAs) and byproducts (such as SBAs and lipopolysaccharides, LPS). The binding of SBAs-TGR5 or LPS-TLR4 (Toll-like receptor 4) can activate UCP1 expression in BAT, and stimulate local type 2 deiodinase (D2) to induce the conversion from the prohormone thyroxine (T4) to bioactive T3 as well. The binding of NE with *β*1,2,3-AR in white adipose tissue (WAT) activates lipolysis (catabolism of triglycerides) into free fatty acids as one source of fuel for BAT thermogenesis, leading to the decrease in body fat content. Cold-induced glucose uptake via glucose transporter (GLUT4) in adipocytes and muscle can also provide glucose as fuel for BAT and muscle thermogenesis. Muscle NST is mainly based on activities of sarcoplasmic reticulum ryanodine receptor (RyR)-mediated Ca^2+^-leak, sarcolipin (SLN)-activated sarcoplasmatic reticulum Ca^2+^-ATPase (SERCA), and mitochondrial UCP3 as well. SLN, via binding to SERCA, can uncouple ATP hydrolysis from Ca^2+^ transportation and as a result increase heat production.

Besides BAT-dependent thermogenesis in eutherian mammals, increasing evidence has shown that skeletal muscle also contributes importantly to cold-induced adaptive thermogenesis, independent of shivering or contraction (Nowack et al., 2017; Bal et al., 2018; Bal and Periasamy, 2020). The role of skeletal muscle NST is particually crucial in several specific endothms, such as birds, monotremes, marsupials, and wild boars, which lack BAT (Rowland et al., 2015). Lineage tracing studies have revealed that skeletal muscle and BAT arive from the same precursor cells in the dermomyotome expressing Pax3/7 (the transcription factor) and myogenic factor 5 (Myf5, the determination factor) (Lang et al., 2005; Sgaier et al., 2005), whereas the precursor for mature white adipocytes lacks Myf5 expression (Sanchez-Gurmaches et al., 2012). Moreover, recent evolutionary studies imply that NST in skeletal muscle originated much earlier than BAT-driven thermogenesis in vertebrates (Rowland et al., 2015; Grigg et al., 2022). The mechanism for muscle NST is based on activities of sarcoplasmic reticulum ryanodine receptor (RyR)-mediated Ca^2+^-leak and sarcolipin (SLN)-activated sarcoplasmatic reticulum Ca^2+^-ATPase (SERCA) (Figure 1) (Periasamy and Huke, 2001; Zima et al., 2010; Bal et al., 2012; Chambers et al., 2022). SLN, via binding to SERCA, uncouples ATP hydrolysis from Ca^2+^ transportation and as a result increases heat production (Smith et al., 2002; Bal et al., 2012). In addition, the activation of mitochondrial UCP3 by intracellular levels of Ca^2+^ and fatty acids is also associated with muscle NST (Costford et al., 2007). Small mammals depend on increasing the adaptive thermogenesis deriving from BAT and skeletal muscle to maintain *T*_b_ relatively stable and survive in harsh winter.

### Thermo-TRPs—SNS—Gut Microbiota—Thermogenesis Axis

Cold signal is sensed by a group of cold-sensitive thermoreceptors, the thermosensitive transient receptor potential channels (Thermo-TRPs), such as TRPA1 (<17°C) and TRPM8 (10–26°C), on the skin and in muscle, viscera, spine and brain, and transmitted through the sensory afferent nerve to the preoptic area (POA) in the hypothalamus and other thermoeffector efferent circuits (Morrison and Nakamura, 2019). There are also warm-sensitive thermoreceptors, such as TRPM2 (>42°C), TRPV1 (>42°C) and TRPV2 (>52°C) on the skin and body core (Hoffstaetter et al., 2018). These TRPs are activated by high *T*_a_ and transfer thermal signal to the central relevant thermoregulatory network and, consequently, lead to BAT atrophy and heat dissipation (Vandewauw et al., 2018). A rescent study identified the expression of TRPV1 as a specific characteristic of vascular smooth muscle (VSM)-derived adipocyte progenitor cells and the contribution of these cells to cold-induced BAT recruitment (Shamsi et al., 2021). TRPV1 is also involved in temperature preference behavior-associated thermoregulation in Mongolian gerbils (Wen et al., 2021). TRPs have been widely studied for cold or warm sensation in specific TRP knock-out laboratory models (Peier et al., 2002; Vandewauw et al., 2018), but the responses of TRPs to *T*_a_s are diversified in wild mammal species and in diverse tissues. For example, during cold acclimation, the expression of TRPM8 in small intestine increased in mice (Wen et al., 2020), and TRPA1 and TRPV2 in BAT increased in Brandt’s voles, whereas TRPM2 decreased in Mongolian gerbils (unpublished data). TRPs can also be activated by ligands, such as capsaicin, menthol and bacterial endotoxins (lipopolysaccharides, LPS) (Peier et al., 2002; Baskaran et al., 2016; Boonen et al., 2018). Therefore, the functions of diverse TRPs should be widely investigated for better understanding how small mammals sense and tolerate extreme *T*_a_ particularly under the ecological contexts of heat waves and extremely cold weather.

Besides the central mechanism of thermoregulation, the peripheral gut microbial community is also involved in the regulation of host metabolic and thermal homeostasis. Several wild studies indicate that gut microbes vary with seasons, altitudes and geographies in small mammals (Maurice et al., 2015; Perofsky et al., 2019). The environmental factors such as diets, *T*_a_ and photoperiod influence the gut microbial community (Martinez-Mota et al., 2019; Suzuki et al., 2019; Khakisahneh et al., 2020). Our previous data identified that low *T*_a_ can directly drive gut microbial community assembly rather than cold-induced feeding effect, and intermittent fluctuations of low and high *T*_a_ altered the diversity and composition of gut microbiota (Bo et al., 2019; Khakisahneh et al., 2020). Cold-induced alteration in microbes induced increases in NE concentrations both in the intestine and BAT, and also increases in concentrations of bacterial metabolites and byproducts, such as SCFAs and SBAs in feces and blood (Worthmann et al., 2017; Bo et al., 2019). Consequently, these metabolites and byproducts, via their respective G protein-coupled receptors (such as GPR43/41, also named FFAR2/3) and the Takeda GPR (TGR5), promote host’s adaptive thermogenesis both in BAT and skeletal muscle (Figure 1). Besides, the increase in NE production could conversely induce reshaping of the gut microbial community, which was verified by pharmacological NE manipulation (Bo et al., 2019). In addition, early evidence has shown that both the microbiota and their hosts produce the same neuroactive compounds (such as pheromones, hormones and neuropeptides) and the receptors, which enable bidirectional communications between the hosts and symbiotic microorganisms (Roth et al., 1982; Lyte, 2014). Therefore, the interaction between host neurotransmitters and gut microbes contributes to host thermoregulation and thermal homeostasis under the changing environments. Cumulatively, available experimental data suggest that the disturbance of gut microbial community may be a mechanism by which changing global temperatures are imposing impacts on fitness of animals in wild-living populations.

### Thyroid—Gut Microbiota—Thermogenesis Axis

Thyroid hormones, particularly the bioactive tri-iodothyronine (T3), have important roles in regulating developmental and metabolic processes (Mullur et al., 2014; Ortiga-Carvalho et al., 2014). The intracellular T3 in tissues and serum T3 are generally derived from the prohormone thyroxine (T4) through the catalytic action of type 2 deiodinase (D2). D2 is highly expressed in the brain, pituitary, and peripheral tissues such as BAT and skeletal muscle (Brent, 2012; Mullur et al., 2014). When bound to the nuclear receptors (α and β) of thyroid hormones, T3 can regulate multiple transcriptional expressions such as adipose triglyceride lipase (Atg5), COX, UCP1 and GLUT4 responsible for fatty acid oxidation, heat production and glucose uptake in BAT (Mullur et al., 2014; Yau et al., 2019). In muscle, T3 can also modulate the expression of GLUT4, SERCA and UCP3 (Solanes et al., 2005; Salvatore et al., 2014). Thyroid hormones are tightly controlled and maintained within a narrow range of circulating levels by a negative feedback mechanism involving hypothalamic thyrotropin-releasing hormone (the hypothalamic-pituitary-thyroid axis, HPA axis) (Ortiga-Carvalho et al., 2014).

The external environment cues induce changes in internal hormones such as thyroid hormones, leptin (adipocytokine) and secretin (gut hormone), which can act as regulators for metabolism and thermogenesis (Zhang and Wang, 2006; Zhao and Wang, 2006; Li and Wang, 2007; Li et al., 2018; Khakisahneh et al., 2019). The circulating T3 and/or T3/T4 levels increased in winter and during low *T*_a_ or short-day acclimations in many mammal species such as in Brandt’s voles, Mongolian gerbils, Daurian ground squirrels (*Spermophilus dauricus*), and plateau pikas (Li et al., 2001). Cold-induced increased levels of serum T3 promote obligatory (BMR) and regulatory thermogenesis (rNST) by stimulating mitochondrial biogenesis, generating and maintaining ion gradients, increasing ATP production, and increasing the expression of uncoupling proteins (UCPs) in liver, muscle and BAT (Mullur et al., 2014; Shu et al., 2017). Additionally, UCP1-independent thyroid thermogenesis was established in UCP1-ablated mice (Dittner et al., 2019), implying the compensatory role of skeletal muscle thermogenesis for cold defense in small mammals.

In addition to the direct effect of T3 on metabolism and thermogenesis, thyroid function is closely related to the homeostasis of intestinal microbial community. The pathologically, chemically or surgically induced thyroid dysfunction (such as hyperthyroidism or hypothyroidism) was associated with alterations in the diversity and composition of gut microbiota (Khakisahneh et al., 2022; Shin et al., 2020). For example, hyperthyroidism in human was followed by enrichments in gut pathogenic bacteria such as *Clostridium* and Enterobacteriaceae (Zhou et al., 2014). And the hyperthyroid gerbils exhibited increased abundances of pathogenic *Helicobacter* and *Rikenella*, and decreased abundances of beneficial *Butyricimonas* and *Parabacteroides* (Khakisahneh et al., 2022). Moreover, the previous data verified that microbiota transplant from normal donors to hyperthyroidism could buffer hyperthyroid-induced disorders in the microbial community and thermogenesis (Khakisahneh et al., 2022). Gut microbes through activation of D2 in the intestine and liver improve the conversion of bioactive T3 from inactive T4, thereby modulating local T3 levels and intestinal homeostasis (Kojima et al., 2019). The gut microbiota-derived SBAs can also activate D2 activity through TGR5 and increase cold-induced thermogenesis (Figure 1) (Watanabe et al., 2006; Worthmann et al., 2017). Moreover, bacterial LPS through binding Toll-like receptor 4 (TLR4) can stimulate T3 conversion by promoting D2 mRNA accumulation and enzyme activity in mouse mediobasal hypothalamus and rat astrocytes (Boelen et al., 2004; Lamirand et al., 2011). Collectively, the gut microbiota can sense hormones, and regulate thyroid hormone recycling and metabolism through bacterial metabolites and byproducts to modulate deiodinase activity (Virili and Centanni, 2015; Virili and Centanni, 2017; Lopes and Sourjik, 2018). Small mammals maintain metabolic and thermal homeostasis through the bidirectional communication between the thyroid and gut microbiota.

### Huddling and Gut Microbiota

**FIGURE 2:**
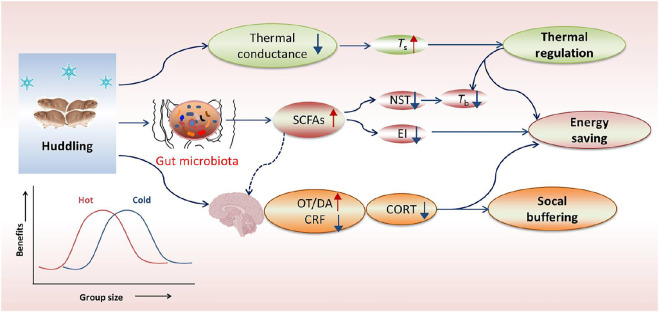
Overview of huddling-related benefits in thermoregulation, energy saving and social buffering. In cold, the gregarious (social) rodents huddle together to reduce body surface area and thus decrease thermal conductance, but can keep a relatively high body surface temperature (*T*_s_) and a low core body temperature (*T*_b_). The social environment can facilitate gut microbial community with more beneficial bacteria and their metabolites such as short-chain fatty acids (SCFAs), thereby inducing decreases in nonshivering thermogenesis (NST) and energy intake (EI). Additionally, social environments particularly social bonding or attachment can also buffer stress responses, which is mediated by increased neurotransmitters such as dopamine (DA) and oxytocin (OT), but reduced corticotropin-releasing factor (CRF) in the limbic system and hypothalamus. The energetic benefit of huddling is dependent on group size and ambient temperatures (*T*_a_), and it indicates a reversed U-shaped form, with an optimal group size for energy saving and other benefits.

The group-living (gregarious or social) mammals have evolved cooperative (or social) behavior to optimize their fitness to survive in harsh environments. Huddling behavior is one type of highly-conserved cooperative behavioral strategies for social species to reduce thermoregulatory costs as well as predation risk (Ebensperger, 2001; Haig, 2008; Gilbert et al., 2010). In cold conditions, the gregarious rodents huddle together to reduce thermal conductance, and decrease NST and food intake, and as a consequence show an increased body surface temperature (*T*_s_), but a reduced *T*_b_ (Figure 2), whereas the solitary species or non-huddling individuals increase thermogenesis to maintain *T*_b_ or entering hibernation to reduce temperature difference between the body and air (Nowack and Geiser, 2016; Sukhchuluun et al., 2018; Zhang et al., 2018). The energetic benefit of huddling depends on group sizes and *T*_a_ in a reversed U-shaped form (Figure 2), and the optimal group size for energy saving varies with different temperatures (Gilbert et al., 2010). Increasing group size over the optimal size has little additional advantage to reduce energy metabolism, but indeed increases resource competition and disease risks among individuals (Gilbert et al., 2010; Škop et al., 2021). In addition, the benefit and intensity of huddling increase as *T*_a_ reduces, and decrease at high *T*_a_ and within TNZ (Gilbert et al., 2010). In conclusion, huddling is an active, complex social thermoregulation behavior, particularly beneficial for energy conservation for social species living in winter or exposing to low *T*_a_.

Social environment (or sociability) can buffer stress responses and facilitate the gut microbial community. For example, social bonding or attachment in the prairie vole (*Microtus ochrogaster*) reduces stress hormone corticosterone (CORT) and confers social buffering benefit, which is mediated by increased neurotransmitters such as dopamine (DA) and oxytocin (OT), but reduced corticotropin-releasing factor (CRF) in the limbic system and hypothalamus (Figure 2) (Smith and Wang, 2014; Burkett et al., 2016). Our previous data in Brandt’s voles showed that huddling animals had a healthier gut microbial community with more SCFAs-producing bacteria in comparison with non-huddling voles, indicating the energy-saving benefit of huddling is mediated by improving microbial homeostasis (Figure 2) (Zhang et al., 2018). Social status also shaped the diverse microbial community between dominants and subordinates in mice and *Tscherskia triton* (Zhao et al., 2020; Agranyoni et al., 2021). Whether gut microbial community mediates sociability-induced physiological and behavioral differences was validated by fecal/caecal microbiota transplant (FMT/CMT) using antibiotics-treated or germ-free mice or wild rodents. The mice with absence of gut microbiota elevated the level of corticosterone (the stress hormone) and displayed social behavioral deficits during the assessment of reciprocal social interactions (Buffington et al., 2016). In addition, normal microbiota colonization (co-housing or oral gavage) could effectively correct social impairments of the mice lacking the gut microbiota (Buffington et al., 2016; Chu et al., 2019). More importantly, a specific bacterial species, *Enterococcus faecalis*, has been identified to restrain stress responses and promote social activity in the mice following social stress (Wu et al., 2021). These substantial data support crucial functions of the gut microbiota in regulating sociability-related behavioral divergence and fitness benefits.

### Coprophagy and Gut Microbiota

**FIGURE 3:**
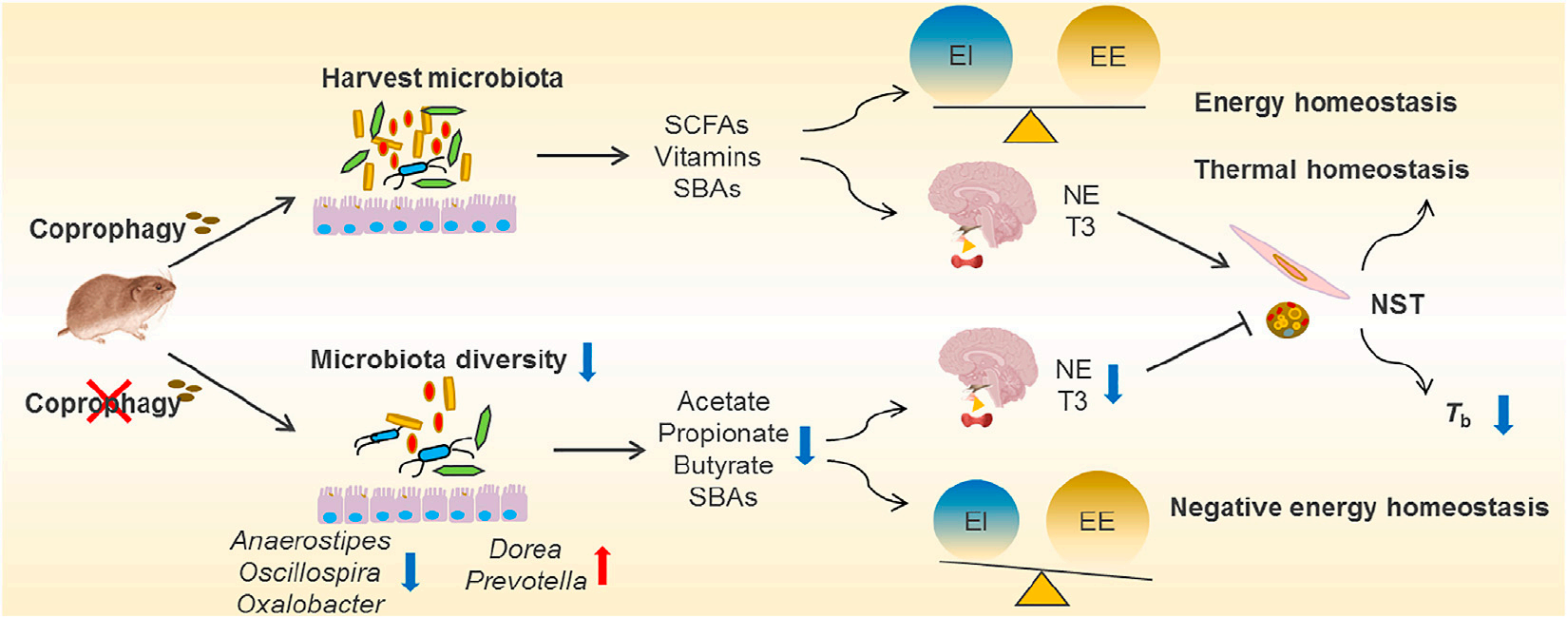
Overview of coprophagy-associated benefits in harvesting intestinal microbiota and maintaining energy and thermal homeostasis. Coprophagy prevention (CP) leads to loss of beneficial bacteria such as *Anaerostipes, Oscillospira* and *Oxalobacter* for production of short-chain fatty acids (SCFAs, such as acetate, propionate and butyrate), vitamins and secondary bile acids (SBAs), and results in the negative energy homeostasis (imbalance between energy intake, EI, and energy expenditure, EE). The deficiency in energy supply to the brain impairs the neuroendocrinology and leads to decreases in norepinephrine (NE) and tri-iodothyronine (T3) in CP animals, consequently leading to decreases in nonshivering thermogenesis (NST) in BAT and muscle and a drop in core body temperatures (*T*_b_).

Coprophagy is the behavior of eating feces to help establish the intestinal microbiota, and to acquire protein and micronutrients, which is common in lagomorphs, rodents and other species (Soave and Brand, 1991). The term caecotrophy refers specially to soft faeces ingestion practiced by rabbits and small herbivores (small hindgut fermenters) (Hoernicke, 1981). The proximal colonic separation mechanism observed in many small herbivores can allow for retrograde fluid transport of digesta including bacteria from the proximal colon back into the caecum (Pei et al., 2001; Liu et al., 2007; Hagen et al., 2016). The accumulation of microorganisms in the caecum contributes to a high fermentation rate for bacterial synthesis of nutrients (Karasov et al., 2011). The high-fiber diets promote digesta retention time and production of more soft feces, and coprophagy increases in the periods with a higher energy demand, such as during pregnancy and lactation (Ebino et al., 1988; Hejcmanová et al., 2020). In many studies in rodents such as rats, mice, *L. brandtii* and *Microtus fortis*, prevention of coprophagy decreased body weight, and RMR and NST, suggesting that coprophagy contributes to energy balance and thermoregulation (Sukemori et al., 2003; Sassi et al., 2010; Kohl et al., 2014; Liu et al., 2016; Bo et al., 2020). The energetic and thermal benefits of interspecific coprophagy for survival were observed in plateau pikas that eat yak feces to supplement energy intake in winter (Speakman et al., 2021). Besides, coprophagy in the voles increases neurotransmitters in the hypothalamus and hippocampus, and promotes cognitive performance (Bo et al., 2020). These data indicate that consumption of enough soft feces for herbivorous rodents is necessary to provide enough amounts of nutrients and energy for growth, thermoregulation, and neurodevelopment as well (Figure 3).

Supplement of nutrients and energy via coprophagy may be through harvesting the gut microbiota (self-inoculation). Prevention of coprophagy in Brand’s voles reduced *α* diversity of gut microbial community and altered the composition of the gut microbiota, particularly decreased relative abundance of Firmicutes, including the families of Clostridiaceae, Lachnospiraceae and Ruminococcaceae, which all belong to highly cellulolytic bacteria (Bo et al., 2020). Accompanied by the reduced diversity of gut microbial community, coprophagy prevention also decreased the concentrations of bacterial metabolites, such as acetate, propionate and butyrate (Figure 3). Both the microbial community and the concentrations of SCFAs recovered after the voles were allowed coprophagy. Other evidence showed that the stomach of woodrats (*Neotoma* spp.) and mice harboured highly similar microbial community to large intestine, and the non-coprophagic mice altered the composition of microbiota and the profile of bile acid pool in the small intestine, but no changes were observed in the large intestine, indicating persistent self-reinoculation of microbiota via coprophagy (Kohl et al., 2014; Bogatyrev et al., 2020). In addition, supplementation of acetate reversed energetic, thermal and microbial homeostasis, and rescued neurological and cognitive deficits induced by coprophagy prevention in voles (Bo et al., 2020). However, it is worth mentioning that coprophagy may induce potential disease risk, since some pathogenic bacteria may be transmitted into the intestine from the feces or environments. All these findings reveal that coprophagy in herbivores promotes the stabilization of gut microbial community, and contributes to maintaining host energy balance, thermoregulation and neuronal function.

## Conclusion and Perspectives

Animals and plants are actually holobionts which consist of their own genes and those of symbiotic microorganisms as well. The developments of sequencing instruments, omics technology and bioinformatics advance our understanding of these microorganisms. Growing evidence indicates that bidirectional communication between the gut microbiota and animal organisms mediates host metabolic and thermal homeostasis in wild small mammals for environmental adaptations, which were summarized in this review. Besides, these microorganisms are involved in regulating host water and salt homeostasis (Nouri et al., 2022), immunity and disease defense (Rosshart et al., 2017), as well as population dynamics (Li et al., 2019; Li et al., 2020; Li et al., 2021) in wild-living populations. All in together, the coevolution of host-microorganism symbionts promotes individual survival, population maintenance, and species coexistence in the ecosystems with complicated, variable environments.

More studies should be performed to explore the contributions of BAT and muscle in cold- and diet-induced thermogenesis. Also, the mechanisms by which microbial metabolites and byproducts regulate thermal sensation, neural conductance, and BAT and muscle thermogenesis need to be uncovered for an integrative understanding of thermoregulation in endotherms. Future studies will still focus on much deeper investigations of the functions of specific microbes from the microbial community in wild mammals and try to screen out beneficial microbiota candidates for the therapeutic potentials. Moreover, we will benefit from the combined techniques of culturomics, gene engineering and editing to identify unknown microbes, and operate targeted genetic modifications of specific intestinal microbes for improving resistance to viral and bacterial infections. In addition, further research will be strengthened to investigate the interaction and coevolution between gut microbiomes and their hosts in ecological and evolutionary contexts. As human activity perturbations and climate changes aggravate in magnitude, frequency and duration, and as the uses of antibiotics, pesticides and food additives grow, understanding their profound impacts on the real-time adaptation and long-term evolution of host–microorganism interactions will be getting more and more beneficial and have broader implications on biodiversity protection and future disease therapy.

## Data Availability

The raw data supporting the conclusion of this article will be made available by the authors, without undue reservation.
